# Web survey during COVID-19 pandemic in São Paulo state: how are medical students sleeping and living?

**DOI:** 10.5935/1984-0063.20220066

**Published:** 2022

**Authors:** Karen Wellen da Silva Beltrame, Ivy Kiemle Trindade-Suedam, Sergio Henrique Kiemle Trindade, Maria Noel Marzano-Rodrigues

**Affiliations:** 1 Hospital for Rehabilitation of Craniofacial Anomalies, University of São Paulo, Bauru campus, Sleep Studies Unit, Laboratory of Physiology - Bauru - São Paulo - Brazil; 2 Bauru School of Medicine, Bauru School of Dentistry, University of São Paulo, Bauru campus, Biological Sciences - Bauru - São Paulo - Brazil; 3 Bauru School of Medicine, Bauru School of Dentistry, University of São Paulo, Bauru campus, Department of Pediatric Dentistry, Orthodontics and Public Health - Bauru - São Paulo - Brazil

**Keywords:** Sleep, Sleepiness, Education, Medical, Undergraduate, COVID-19

## Abstract

**Objectives:**

This study aimed at characterizing medical students’ sleep and life quality during the COVID-19 pandemic in São Paulo (SP) state.

**Material and Methods:**

All public higher education institutions (HEIs) of SP state were invited to participate. From a list of 56 private HEIs, 16 were randomly selected. The web survey collected: sociodemographic data; factors related to COVID-19; sleep self-assessment; scores in the Epworth sleepiness scale, Pittsburgh sleep quality index, and student and resident life in the health area - questionnaire.

**Results:**

The HEIs’ acceptance rate was 25% (8), resulting in 200 participants (response rate 5.04%), aged ≥18 years, 60.5% females. Concerning COVID-19, 89.00% never showed symptoms and/or tested positive, 82.00% declared full adherence to epidemiological measures to prevent the infection’s spread, and 45.00% completed the vaccination schedule against SARS-CoV-2. Sleep deprivation was accompanied by a drop of self-perceived sleep quality from 8 to 6 (in a Likert scale) during COVID-19 pandemics (*p*≤0.0001), 76.50% were poor sleepers, and 40.00% had drowsiness, especially women (*p*≤0.05). They also had lower quality of life and unfavorable psychological and physical outcomes than men (*p*≤0.05). Internship students had a more negative perception of the educational environment (*p*≤0.05), characterized by an excessive workload.

**Discussion:**

Women and internship students are a representative fraction that requires special attention and focused strategies to cope with sleep problems and medical education during COVID-19 pandemics.

## INTRODUCTION

Medical students present high rates of poor sleep quality and related disorders above those expected for their age group^1-3^. Among the factors associated with sleep problems in students, the academic overload inherent to medical training^4^, the poor quality of life in the academic environment^5-8^, and sedentary lifestyle^9,10^ stand out.

Neglecting sleep problems by a lack of change in habits and an absence of early or effective diagnosis might put these students at risk, negatively affecting their health^3^. In this population, poor sleep quality has been associated with exacerbation of mental stress, psychosocial suffering, low levels of quality of life, chronic fatigue, daytime sleepiness, and impaired academic performance^7,11-14^.

During the pandemic caused by coronavirus disease 2019 (COVID-19), social distancing measures were imposed to control the spread of the severe acute respiratory syndrome coronavirus 2 (SARS-CoV-2 virus). As a result, among the countless consequences resulting from the health crisis, there was an abrupt interference in the educational process of university education worldwide^15,16^.

Concerns about the impact of the pandemic on the training of future physicians permeate several aspects of medical students’ life and health. COVID-19-related fear and social distancing have been critical sources of anxiety, depression, sedentarism, increased alcohol and tobacco consumption, and impaired sleep quality^17-22^. Despite little controversy^1,2^, most studies suggest that female medical students and those at higher years of medical school had the poorer outcomes concerning sleep and quality of life, in general^3-7^, and during COVID-19 pandemics^11^. Therefore, suggesting that inequities related to gender and educational environment may affect the physical and psychological health status of the students.

This study aimed at assessing the quality of sleep and life of medical students and its relationship with the COVID-19 pandemic through a cross-sectional and observational web survey conducted in the state of São Paulo.

## MATERIAL AND METHODS

### Bioethical considerations

Individual approval was obtained from the Research Ethics Committees of the Hospital for Rehabilitation of Craniofacial Anomalies, University of São Paulo - Bauru campus (CAAE: 40726720.4.0000.5441 and Protocol number: 4.468.963). In addition, before the beginning of the study, an informed consent form was signed by each participant.

### Participating institutions

The National Registry of Higher Education Courses and Institutions e-MEC was used for the primary identification of medical programs^8^. Of the 72 medical school programs in medicine active in the state of São Paulo, 16 are public and 56 are private. Therefore, to obtain an equivalent number of public and private institutions, 16 private HEIs out of 56 were randomly selected.

Contact with the heads of the 32 selected HEIs was made via email at two different times with intervals of 15 days between them. The final response rate was of the invited HEIs that agreed to participate was 25% (8). They were included in following order: 1) São Camilo University Center, São Paulo campus; 2) Faculty of Medicine of Marília; 3) University of Franca; 4) University of São Paulo, Bauru campus; 5) University of São Paulo, Ribeirão Preto campus ; 6) University of Western São Paulo, Jaú campus; 7) São Paulo State University Júlio de Mesquita Filho, Botucatu campus; 8) Federal University of São Carlos. All medical students older than 18 years were eligible for participation and received an email invitation. Those with severe or uncontrolled cardiovascular, metabolic, neurologic, or endocrine illnesses were excluded. It is important to underscore, that users of alcoholic beverages and hypnotics were not excluded, regardless their effects on sleep. Both variables were assessed either through the sociodemographic questionnaire or psychometric scales.

### Data collection

The instrument for data collection was organized in four stages: 1) sociodemographic data^5,8^; 2) survey of factors related to COVID-19^15^; 3) brief sleep self-assessment before and during the pandemic^20,23^; 4) self-application of psychometric scales in the public domain, validated for use in Portuguese - Epworth sleepiness scale (ESS-BR)^24,25^, Pittsburgh sleep quality index (PSQI-BR)^26,27^ and student and resident life in the health area - questionnaire (VERAS-q)^5,8^.

The cutoff points for the interpretation of psychometric scales followed the recommendations in the literature. The ESS-BR score ranged from 0 to 24, and can be interpreted as follow: lower normal daytime sleepiness (0 to 5), higher normal daytime sleepiness (6-10), mild excessive daytime sleepiness (11-12), moderate excessive daytime sleepiness (13-15), and severe excessive daytime sleepiness (≥16) ^24,25^. The PSQI-BR has seven domains, each scored 0 to 3, that can be summed up to a general score equal to 21. The cutoff for poor sleep quality is >5^26,27^. VERAS-q is a 45-item instrument, divided into four domains, whose scores are summed to obtain the final score. The greater the scores, the greater the quality of life. Results are analyzed through comparisons between or among groups^5,8^.

The consent form and the selected psychometric questionnaires were combined into a single instrument, elaborated in the Google Forms tool. It was distributed through the secretariats of the participating courses. The subjects received written instructions to respond to the survey. The estimated time required to complete the survey was 25 minutes, and the deadline for return was 15 days. The instrument distribution was repeated once, 30 days after the first deadline. Data collection occurred between 03/01/2021 and 05/31/2021, during the most restrictive phase of the São Paulo Plan.

### Analysis

The description of continuous variables was made by mean ± standard deviation (SD) or by medians and their respective interquartile ranges, according to normal (Kolmogorov-Smirnov analysis) or non-normal distribution, respectively. Categorical data were presented as frequencies and percentages.

The sample was stratified in two ways, except for sociodemographic data: 1) according to medical course level - I) basic cycle - first and second year, II) clinical cycle - third and fourth year, III) internship; 2) by the declared gender of the participants - I) male, II) female. Considering a type-I error alpha value of 0.05, the sample (N=200) has a power >95% for detecting a magnitude difference of 27% between the mean global scores of PSQI-BR per gender, at the significance level of 0.05, rejecting the null hypothesis (Epi Info^TM^).

Analysis of variance (ANOVA) with Tukey’s posthoc or Kruskal-Wallys with Dunn’s posthoc was applied to compare three samples, parametric or non-parametric, respectively. The two-tailed t-test for independent samples or the non-parametric version of Mann-Whitney was used to verify differences between two means or medians, respectively. The paired t-test or Wilcoxon test was selected to compare paired data. Pearson’s correlation coefficient (r) was used to assess the correlation between the continuous scores (ESS-BR, PSQI-BR, and VERAS-q questionnaires) and ordinal variables. Fisher’s exact test was applied to detect significant associations between categorical data. Confidence intervals (CI) and their lower and upper limits for all analysis were set at the 95% level, and values of *p*≤0.05 were considered statistically significant.

## RESULTS

### Sociodemographic data

Of the 3,966 students who were invited to participate in the survey, 200 agreed to be included, representing a response rate of 5.04%. The sample consisted of 174 (87.00%) students from public HEIs and 26 (13.00%) from private HEIs, with a predominance of female participants (121 or 60.50%) and an age range between 22 and 41 years (median = 22 years). Table 1 presents the description and comparative analysis of the sociodemographic characteristics of the sample.

**Table 1:** Sociodemographic characteristics of the studied sample (N=200).

Variables	Course level						HEI			
	Basic cycle (n=84)		Clinical cycle (n=98)		Internship (n=18)		Public (n=174)		Private (n=26)	
	f	*%*	f	*%*	f	*%*	f	*%*	f	*%*
**Age**										
<25 years	74^*^	88.10	82^*^	83.67	12^*^	66.67	146	83.90	22	84.61
≥25 years	10	11.90	16	16.33	6	33.33	28	16.10	4	15.39
**Gender**										
Masculine	33	39.29	42	42.86	3	17.54	73	41.95	5	19.23
Feminine	51	60.71	56	57.14	14	82.35	101^*^	58.05	21^*^	80.77
**Social programs**										
Not participant	56	66.67	54	55.10	13	72.22	104	59.77	19	73.08
Participant	28	33.33	44	44.90	5	27.78	70	40.23	7	26.92
**Residence**										
Unaccompanied	38	45.24	51	52.04	10	55.56	89^*^	51.15	10^*^	38.46
Accompanied	46	54.76	47	47.96	8	44.44	85	48.85	16	61.54
**Regular smoking**										
No	79	94.05	90	91.84	15	83.33	161	92.53	23	88.46
Yes	5	5.95	8	8.16	3	16.67	13	7.47	3	11.54
**Regular drinking**										
No	30	35.71	41	41.84	8	44.44	65	37.36	14	53.85
Yes	54	64.29	57	58.16	10	55.56	109	62.64	12	46.15

Notes: HEI = Higher education institution; Basic cycle = First and second years of medical course; Clinical cycle = Third and fourth years of medical course; Internship = Fifth and sixth years of medical course; *f* = Frequency; Mann-Whitney U test and the Kruskal-Wallis test with Dunn’s post hoc (^*^p≤0.05).

### Self-perception of health condition related to COVID-19

During the period of the research, 178 (89.00%) participants were healthy (never presented symptoms of the infection, and/or tested positive) concerning COVID-19, 19 (9.50%) had the respiratory infection but were cured, and three (1.50%) had an unconfirmed suspicion of the disease (lacking confirmatory diagnosis). The data indicated that 92 (46.00%) students could identify contact with someone confirmed to be infected with SARS-CoV-2. That was more common among internship students, whose contact with patients occurs more often than in the clinical cycle (*p*≤0.05). There was a statistically significant association between health status (healthy or cured/suspected of COVID-19) vs. identification of the previous contact with a proven infected individual (*p*<0.0001). Fear of illness was reported by 182 (91.00%) participants.

The majority, 164 (82.00%) students declared full adherence to epidemiological measures to prevent the spread of COVID-19. Almost half of the sample (90 or 45.00%) completed the vaccination schedule against COVID-19; 26 (13.00%) took the first dose and the remainder (84 or 42.00%) had not been immunized but intended to be vaccinated. The number of vaccinated students in the internship level was statistically higher than in the basic cycle (*p*≤0.05), with no difference between public and private HEIs.

### Sleeping during the pandemic

#### Sleep self-assessment: quality, quantity, and regularity

The median of continuous sleep time considered ideal for feeling rested was 8 hours (25^th^ percentile = 8; 75^th^ percentile = 9). The median nighttime sleep duration equal to 7 hours (25^th^ percentile = 6; 75^th^ percentile = 8) indicated sleep deprivation. In the period evaluated, students exhibited variability in the amount of nighttime sleep during the week (*p*<0.0001) and on weekends (*p*<0.0001) when subjectively compared to the pre-pandemic period. Most slept less than usual, both on weekdays and on weekends: 44.00% (88) and 54.50% (109), respectively. In addition, sleep habits showed irregularity, with 66.50% (133) of the participants going to bed later and 66.0% (132) getting up later than before the pandemic (*p*<0.0001).

Self-perceived sleep quality measured on a 10-point Likert scale, subjectively comparing sleep before and during the pandemic, dropped at a statistically significant level (*p*<0.0001) from 8 (25^th^ percentile = 6; 75^th^ percentile = 8) to 6 (25^th^ percentile = 4; 75^th^ percentile = 8) (Graph 1). It was not possible to correlate sleep regularity or duration with self-perceived quality. However, this parameter correlated strongly and negatively with the PSQI-BR (r=-0.769; 95%CI = -0.703 to -0.821; *p*<0.0001).

#### Psychometric data on sleep quality and daytime sleepiness

The description of the data obtained through the PSQI-BR and ESS-BR and their comparison between the sexes and by course level - the only sociodemographic variables that showed a statistically significant relationship with the psychometric tests - is shown in Table 2. The sample had poor sleep quality in 76.50% of the cases, and females had a higher overall score in the PSQI-BR than males (*p*≤0.05).

**Table 2:** Median values and interquartile range (25%-75%) of sleep quality of medical students, according to the PSQI-BR and ESS-BR, stratified by gender and course level.

Instrument	Gender				Course level						Total (N=200)	
	Masculine (n=78)		Feminine (n=122)		Basic cycle (n=85)		Clinical cycle (n=98)		Internship (n=17)			
**PSQI-BR**												
Global score	6.50^*^ (5.00-9.00)		9.00^*^ (6.00-13.00)		7.00 (6.00-10.75)		7.00 (5.00-10,00)		9.00 (7.00-13.00)		8.00 (6.00-11.00)	
	**f**	***%***	**f**	***%***	**f**	***%***	**f**	***%***	**f**	***%***	**f**	***%***
≤5	16	25.52	15	12.30	19	22.35	27	27.55	1	5.88	47	23.50
>5	62	74.49	107	87.70	66	77.65	71	72.45	16	94.12	153	76.50
**ESS-BR**												
Global score	8.00^*^ (4.75-11.00)		12.00^*^ (8.00-15.00)		10.00 (7.00-15.00)		9.00 (5.00-13.00)		12.00 (6.50-16.50)		9.00 (6.00 - 14.00)	
	**f**	***%***	**f**	***%***	**f**	***%***	**f**	***%***	**f**	***%***	**f**	***%***
≤10	59	75.64	57	46.72	35	41.67	39	39.80	9	50.00	119	59.50
>10	19^*^	24.36	65^*^	53.28	49	58.33	59	60.20	9	50.00	81	40.50

Notes: PSQI-BR = Pittsburgh sleep quality index in Portuguese; ESS-BR = Epworth sleepiness scale in Portuguese; Basic cycle = First and second years of medical course; Clinical cycle = Third and fourth years of medical course; Internship = Fifth and sixth years of medical course; *f* = Frequency; Data do not follow the normal distribution (Kolmogorov-Smirnov test); Mann-Whitney U test, Kruskal-Wallis test with Dunn’s post hoc test, and Fisher’s exact test (^*^p≤0.05).

When comparing the frequencies of scores between the domains of this instrument, it was observed that score 3 was higher in domains 2 (sleep latency) and 7 (daytime dysfunction) (*p*<0.0001) (Graph 2). Score 3 in domain 2 was reported by 51 females vs. 13 males (*p*≤0.05). It is shown that women experienced a detrimental increase in time between going to bed and initiating sleep three or more times a week. Similarly, domain 7 also grouped more women than men in score 3 (*p*≤0.05), indicating more significant daytime dysfunction due to poor sleep quality.

Among the 200 students evaluated using the ESS-BR, 81 (40.50%) had excessive daytime sleepiness. Of these, 21 (10.50%) had a mild excessive increase (11 to 12 points), 32 (16.00%) moderate excessive increase (13 to 15 points), and 28 (14.00%) severe excessive increase (≥16 points). Females were more affected by sleepiness than males (*p*≤0.05) and had higher global test scores (*p*<0.0001). In addition, a weak positive correlation between poor sleep quality and excessive daytime sleepiness was seen (r=0.355; 95%CI = 0.275 to 0.509; *p*<0.0001), as determined by the PSQI-BR and ESS-BR, respectively.

#### Quality of life and medical education during the pandemic

##### Self-assessment of the teaching-learning process

Most individuals reported self-perceived concentration difficulties (183 or 91.50%) and learning deficits (174 or 87.00%). Limited access to remote academic activities was found in more than a quarter of the sample (52 or 26.00%). Most students reported disruption to their school schedule resulting in delays in academic activities (189 or 94.50%) and inadequacy of pedagogical strategies for implementing online learning (173 or 86.5%). Although 73.00% (146) had already partially resumed face-to-face activities (internship vs. clinical cycle *p*≤0.05; clinical cycle vs. basic cycle *p*≤0.05), 95.50% (191) of the students had to alter their habitual study methods to cope with new paradigms imposed by e-learning.

##### Student quality of life and educational environment

When questioned whether the COVID-19 pandemic negatively impacted their quality of life as medical students, 157 (78.50%) partially or totally agreed and 43 (21.50%) reported indifference, partial disagreement, or total disagreement. The results of VERAS-q are summarized in Table 3.

**Table 3:** Mean values (± standard deviation) of the student’s quality of life, stratified by gender and level of medical course.

VERAS-q	Gender		Course level			Total (N=200)
	Masculine (n=78)	Feminine (n=122)	Basic cycle (n=84)	Clinical cycle (n=98)	Internship (n=18)	
Global score	144.00±23.86^*^	134.12±23.86^*^	142.60±22.74^*^	136.27±25.27	126.10±21.54^*^	140.60±24.50
Domains						
1	35.58±7.61^*^	32.22±7.71^*^	34.76±7.67^*^	33.21±7.89	29.35±6.95^*^	33.54±7.83^*^
2	35.12±8.47^*^	31.49±7.88^*^	34.10±7.70	32.44±8.71	29.76±8.03	32.91±8.29^*^
3	25.23±4.98^*^	23.45±5.25^*^	24.21±5.05	24.32±5.47	22.88±4.49	24.15±5.21^*^
4	48.06±7.55	47.03±8.11	49.49±7.47^*^	46.26±7.82^*^	44.12±8.29^*^	47.44±7.90^*^

Notes: VERAS-q = Quality of life and academic environment quality of health sciences students - questionnaire; Domains: 1 = use of time, 2 = psychological, 3 = physical, 4 = academic environment; Basic cycle = First and second years of medical course; Clinical cycle = Third and fourth years of medical course; Internship = Fifth and sixth years of medical course; Data were normally distributed (Kolmogorov-Smirnov test); Student’s t-test or ANOVA analysis of variance with Tukey post hoc (^*^p≤0.05).

The perception of quality of life was worse in females than in males and among internship students vs. basic cycle ones (*p*≤0.05). As expected, the higher the overall scores for the PSQI-BR (r=-0.643; 95%CI = -0.565 to -0.726; *p*<0.0001) and the ESS-BR (r=-0.337; 95%CI = - 0.255 to -0.493; *p*<0.0001), the lower the quality of life, determined by the global score of the VERAS-q (Graph 3).

Furthermore, a better perception of quality of life correlated moderately and positively with quality of the academic environment (r=0.595; 95%CI = 0.464 to 0.654; *p*<0.0001) and general health status (r=0.567; 95%CI = 0.497 to 0.678; *p*<0.0001). The perception of the correlation of self-care with health (r=0.533; 95%CI = 0.426 to 0.626; *p*<0.0001) and the regular practice of physical exercise (r=0.521; 95%CI = 0.412 to 0.615; *p*<0.0001) had a moderate positive impact on general health status.

The physical domain (3) had the lowest scores in the sample, indicating a more negative effect on the quality of life (*p*≤0.05). The domain of the teaching environment (4), on the other hand, had the highest scores, exerting the most positive effect (*p*≤0.05).

Female students had lower scores than male students in the following domains: time management (1), psychological (2), and physical (3) (*p*≤0.05). When comparing the medical course levels, time management (1) was worse in the internship vs. basic cycle (*p*≤0.05). Students in more advanced years (clinical cycle and internship) also scored lower in the academic environment perception than students of the basic cycle (*p*≤0.05).

Excessive academic activities were moderately and positively correlated with lack of time to study (r=0.500; 95%CI = 0.388 to 0.597; *p*<0.0001), limited availability for extracurricular activities (r=0.528; 95%CI = 0.420 to 0.622; *p*<0.0001), and stress (r=0.518; 95%CI = 0.408 to 0.612; *p*<0.0001). In turn, not having enough free time was related to the lack of time to study (r=0.507; 95%CI = 0.396 to 0.604; *p*<0.0001). The negative impact of the COVID-19 pandemic on the students’ quality of life correlated, however weakly, with a worse time management capacity (r=0.330; 95%CI = 0.197 to 0.451; *p*<0.0001).

The feeling of discouragement reported by 144 (72.50%) students was associated at a moderate level with increased unrealistic self-expectations (r=0.514; 95%CI = 0.403 to 0.609; *p*<0.0001), lack of concentration (r=0.586; 95%CI = 0.487 to 0.671; *p*<0.0001), stress (r=0.579; 95%CI = 0.478 to 0.664; *p*<0.0001), and anxiety (r=0.526; 95%CI = 0.417 to 0.620; *p*<0.0001). The latter was positively correlated with a greater negative impact of COVID-19 on the student’s quality of life (r=0.330; 95%CI = 0.186 to 0.442; *p*<0.0001).

## DISCUSSION

During three months of the most restrictive period of social distancing measures to control the pandemic, 200 students from eight medical schools across São Paulo state in Brazil were surveyed concerning sleep quality, daytime sleepiness, and quality of life. The most critical limitations were as follows: 1) some of the results represent participants’ recalls of sleep habits and quality of life before the pandemic, which can impose natural bias; 2) the cross-sectional study’s design does not allow the establishment of a cause-effect relationship among variables; 3) HEIs’ agreement to participate, and students’ response rate were insufficient for assessing survey validity.

A strength of this study is that we presented data gathered from clinical cross-cultural validated psychometric tests - PSQI-BR and ESS-BR^2,3,25,27,28^. Furthermore, a questionnaire which has been widely applied for measuring the quality of life and academic environment quality of health sciences students - VERAS-q - was used^5,6,8^. Another aspect that should be highlighted refers to the collection of data from multiple medical schools in the state of São Paulo, and not just from our center. Also, those who participated completed the whole instrument without losses, indicating no technical difficulties or significant respondent fatigue.

Main results were as follows: 1) participants’ self-perception was that the COVID-19 pandemic negatively impacted sleep quality, quantity, and regularity, and their quality of life as medical students; 2) most of them were poor sleepers, and nearly half presented drowsiness; 3) females were more negatively affected than males; 4) the undergraduate medical environment is characterized by excessive academic activities, poor management of time, unfavorable psychological and physical outcomes, and poor quality of life.

Other studies that assessed COVID-19 pandemic implications in medical student life also observed acute implications with unknown long-term consequences, with substantial alterations in quantity and quality of sleep^21,29^ and a rise in anxiety and depression^29,30^.

The students presented sleep deprivation, lack of sleep regularity, and overall poor sleep quality during the COVID-19 pandemic. High latency and diurnal dysfunction were detected in the PSQI-BR questionnaire, which showed high continuous scores (8.00) in 76.50% of the sample. The absence of sleep regularity indicates the inadequacy of sleep habits. The fact that students tended to go to bed later and wake up later during the pandemic, suggests a delayed phase shift. Despite being common in the second decade of life, the delayed phase shift during COVID-19 pandemics would possibly be associated with increased homework load and use of electronic devices. In addition, recommended social confinement might contribute to this finding since it promoted altered social timekeepers essential to synchronizing the light-dark cycles and biological responses, favoring burnout and negatively affecting academic performance^31,32^.

Increased sleep latency has been associated with decreased physical activity, blue-light emission devices, and smoking status^33^. Despite these associations not being statistically detected in our sample, the physical activity domains presented the lowest scores in the VERAS-q questionnaire. Most academic activities were performed on electronic devices, which might have caused augmented screen time. Also, increased anxiety and worries before sleep have been associated with increased sleep latency during the COVID-19 pandemic^20^. In our sample, anxiety was associated with a more negative quality of life, being frequently reported as a problem for having disturbed sleep in PSQI-BR, and was also related to the high academic load, possibly justifying these findings.

PSQI-BR scores correlated with drowsiness ESS-BR≥10, as indicated by others^28,34^. The frequency of excessive daytime sleepiness was nearly 40.00% in our sample, as previously reported for medical students^12,35^. Despite the well-established effects of drugs (i.e., hypnotics, anxiolytics, and alcohol) or diseases (i.e., psychiatric disorders) on sleep quality and excessive daytime sleepiness, they were not considered as exclusion criteria in this study. Neither, the influence of these confounding variables was controlled through statistical strategies, such as randomization, restriction, or matching. Although, these characteristics were investigated through the psychometric instruments, to make a better acquittance of the students’ health profile.

Sleepiness severity was not associated with sleep deprivation, increased sleep latency, or hypnotic drug use, which have been considered important contributors to drowsiness in previous reports^34-36^. The use of hypnotics three or more times a week was scarce, and nearly 80% never used pharmacological sleep inductors during the surveyed period, according to the PQSI results (question number 10). Even though there was not association between sleepiness and drinking, we highlight that 121 (60.50%) students were regular consumers of alcoholic beverages, which is a considerably high rate.

It is important to underscore that the STOP-Bang (Snoring, Tired, Observed, Pressure, Body mass, Age, Neck size, Gender) questionnaire was applied in this sample (Supplementary Table 1), and no association between sleepiness and obstructive sleep apnea was found, contrary to other findings^37^. It is possible that the comprehension of sleepiness in this population requires a more profound biopsychosocial study of how these factors act in combination^34^, especially in women, which were more affected than men.

Sex disparities concerning sleep quality scores and sleepiness occurrence were detected in our sample, as verified in previous studies^38-40^. The exact difference in sleep mechanisms between males and females is still a matter of debate. Some evidence suggests that sleep is sensitive to the fluctuation of estrogens and progestins, and sleep complaints overlap with puberty and ovarian cycle^41^. However, to what extent hormonal characteristics would be affected sleep quality during stay-at-home orders, is unclear, in the context of our study. A study has shown that women’s perceived higher anxiety levels due to COVID-19 are associated with sleep quality assessed through PSQI^42^. Therefore, anxiety severity may explain the gender disparity of sleep quality observed in the present study, better than hormonal changes, which are not expected to be affected by the pandemic and/or social isolation.

In this context, it is also essential to consider that women report their symptoms differently than men. For example, female medical students have been considered more critical about their perceptions, while male students tend to underappreciate physical and psychological symptoms^8^. Therefore, biological characteristics and cultural factors should also be considered when interpreting sleep^43^ and quality of life perception^8^. Also, the subjective academic assessment of students and the workforce in academic settings have a bias that disadvantages women, imposing additional challenges that contribute to increase in anxiety and stress, and a decrease in quality of life for females in academic and clinical settings^44^.

The global score of VERAS-q and scoring in time management, psychological, and physical domains were lower in women than men, as previously reported^8^. Time management difficulties were associated with a more significant negative impact of COVID-19 on students’ quality of life. Also, it was observed that the excessive academic workload was associated with both insufficient time for studying and stress, with an increased worsening among internship students. It can be inferred then that self-regulated remote learning is a complex process and adhering to a schedule at home can be challenging^45^. Professors and institutions should be aware of this and assist students in planning their activities, aiming at preventing poor academic performance^46^ and burnout during tele-education. Management of online schedules has been of particular concern for students of underprivileged backgrounds^47^. They are more influenced by environmental factors and inequity in provision and access to remote platforms and technology, a difficulty reported in at least one of every four students in our sample.

Psychological aspects assessed through VERAS-q indicated high rates of discouragement (70%), associated with concentration problems, increased anxiety, stress, and unrealistic self-expectations. Anxiety affects 33.8% of medical students’ worldwide^48^, and this burden persists throughout the medical career. During COVID-19, those with preexistent high levels of stress and anxiety presented worsening in their mental health^49^. Therefore, students must be encouraged to seek psychological attention, and medical schools should contribute to destigmatizing mental illness. Furthermore, action plans must be developed to help students cope with negative perceived implications of psychological treatment upon the medical career, fear of documentation, and concerns about confidentially, thus improving their knowledge and confidence to seek help^48,50^.

Maintaining regular physical activity was associated with a better perception of self-care and overall health quality in our sample. Nevertheless, females reported lower scores in the VERAS-q physical domain than males, which is consistent with previous reports^51^. Increased sedentary behavior in medical students has been reported during the COVID-19 pandemic^19^, leading to deleterious effects on mental health and well-being as well as the prevention of non-communicable diseases^52^. Vancini et al. (2021)^53^ proposed that initiatives to encourage and give access to low-cost home-based exercise programs should be encouraged, primarily through technological resources that might be helpful from a public health perspective during COVID-19.

Finally, it is relevant to discuss a general perception of excessive academic workload, with incompatible free time for studying and extracurricular activities, and associated increased levels of stress. These findings are commonly observed in the assessment of medical students, and according to Damiano et al. (2021)^54^ stress is generally related to an unhealthy learning environment and poor academic performance. The VERAS-q learning environment and time management domains were statistically significantly worse among internship students, as observed in previous studies in Brazil. Causality has been attributed to insufficient educational support, tiredness facing the increase in workload and responsibility, the inadequacy of professors’ and preceptors’ feedback, and increased competitivity for the residence selection processes^8,55^. Questions remain regarding how to adapt medical curricula, provide continuing education of professors and preceptors, help students develop the necessary skills for transitioning from clinical years to internship and medical residence, and increase satisfaction in clinical practice^56^.

Students’ physical and mental health and their perception of academic environment adequacy and quality of life should be systematically evaluated. Aiming to achieve integrality in medical education, HEIs might focus in improving academic, psychological support and well-being, including sleep quality and excessive daytime sleepiness. Reformulation of curricula, improving soft skills development, searching for equality in access to e-learning environments, and continuous qualification of professors must be of great concern. In general, medical students of São Paulo state during COVID-19 qualified as poor sleepers, suffering from drowsiness and having a quality of life that could be improved. Women and internship students are a representative fraction that requires special attention and focused strategies to cope with medical education.

## Supplementary Material

**Supplementary Table 1:** Descriptive data of obstructive sleep apnea risk, assessed through STOP-Bang questionnaire (N=200).

STOP-Bang	Total (N=200)		Gender				Course level						HEI			
			Fem. (n=122)		Masc. (n=78)		Basic cycle (n=84)		Clinical cycle (n=98)		Internship (n=18)		Public (n=174)		Private (n=26)	
	f	*%*	f	*%*	f	*%*	f	*%*	f	*%*	f	*%*	f	*%*	f	*%*
Low risk (0-2)	194	97.00	121	99.18	75	96.15	81	96.43	96	97.96	17	94.44	168	96.55	26	100.00
Intermediate risk (3-4)	1	0.50	1	0.82	0	0.00	0	0.00	0	0.00	1	5.56	1	0.57	0	0.00
High risk (≥5)	5	2.50	2	0.00	3	3.85	3	3.57	2	2.04	0	0.00	5	2.87	0	0.00

Notes: STOP-Bang = Snoring, Tired, Observed, Pressure, Body mass, Age, Neck size, Gender questionnaire; Fem. = feminine; Masc. = masculine; Basic cycle = First and second years of medical course; Clinical cycle = Third and fourth years of medical course; Internship = Fifth and sixth years of medical course; HEI = Higher Education Institution; *f* = frequency; Data do not follow the normal distribution (Kolmogorov-Smirnov test); No statistically significant differences were detected.
